# Sensory Preference and Nutrient Content of Sorghum Substitute Bread With Strawberry Dadih Vla

**DOI:** 10.1155/ijfo/7887215

**Published:** 2026-07-28

**Authors:** Helmizar Helmizar, Jeallyza Muthia Azra, Faza Yasira Rusdi, Yuliana, Healthy Hidayanty, Restu Sakinah

**Affiliations:** ^1^ Department of Nutrition, Faculty of Public Health, Universitas Andalas, Padang, Indonesia, unand.ac.id; ^2^ Culinary Study Program, Department of Family Welfare Science, Faculty of Tourism and Hospitality, State University of Padang, Padang, Indonesia; ^3^ Department of Nutrition Science, Faculty of Public Health, Hasanuddin University, Makassar, Indonesia, unhas.ac.id; ^4^ Nutrition Study Program, Pekanbaru Medical Center Institute of Health Science, Riau, Indonesia

**Keywords:** bread, dadih, nutrient, sensory, sorghum, vla

## Abstract

Sorghum (*Sorghum bicolor* (L.) Moench) is a cereal crop ranked the world′s fifth most important cereal grain after wheat, maize, rice, and barley. Sorghum grain protein varies from 4.4% to 21.1%, with a mean value of 11.4%. Sorghum grain is known for its hardness compared to other food grains. The hardness of the grain is due to a higher content of protein prolamin (3.6%–5.1%). Dadih is a fermented buffalo milk with various lactic acid bacteria (LAB). Dadih could be developed into other products such as vla. This study was aimed at analyzing sensory preference and nutrient content of dadih vla. Dadih was collected from Agam District, West Sumatra. The development of sorghum and dadih substitute bread products was carried out by dividing them into three product formulations, namely, 0%, 5%, and 10% sorghum substitute bread and vla with the addition of 0%, 15%, and 20% strawberries. Based on the physicochemical and sensory tests that have been carried out, it was found that the best formulation was a bread formula with 5% sorghum substitution and 15% strawberry vla. The selected formula contains 39.24% water, 48.51% carbohydrates, 0.76% ash, 5.85% protein, 5.74% fat, 1.81% crude fiber, and 43.89 mg vitamin C. The pH value is 4.89 with a brightness of 56.87, a redness of 5.60, and a yellowness of 16.97. The most abundant amino acid in the sorghum substitute bread (5% with strawberry vla, 15%) was glutamate, followed by proline, aspartic acid, serine, histidine, phenylalanine, glycine, arginine, valine, alanine, threonine, isoleucine, tyrosine, tryptophan, cystine, and methionine. The antioxidant activity of sorghum substitute bread 5% with strawberry vla 15% is 93,667.44 *μ*g/mL. For future research, it is recommended that product testing be carried out in accredited laboratories and that testing of other nutritional variables be conducted so that the resulting product is more optimal and complete.

## 1. Introduction

Sorghum (*Sorghum bicolor* (L.) Moench) is a cereal crop ranked as the world′s fifth most important grain, after wheat, maize, rice, and barley. It contains 8%–18% protein, 70%–80% carbohydrates, 19% dietary fiber, and about 3% crude fiber, as well as various minerals and vitamins. Starch is the main carbohydrate, stored mostly in the endosperm, providing slow‐digesting complex carbohydrates that supply steady energy. The grain also contains fructosan and is harder than other cereals due to its higher prolamin and lysine content. Its proteins, which include albumins, globulins, glutelins, and kafirins, lack gliadin and glutenin, making sorghum a naturally gluten‐free and nutritious grain [1, 2].

According to Mohamed et al. [3], sorghum grain protein content ranges from 4.4% to 21.1%, with a mean value of 11.4%. Sorghum grain is known for its hardness compared to other food grains. The hardness of the grain is due to a higher content of protein prolamin (3.6%–5.1%). The lysine content ranges from 1.06% to 3.64%. The protein fractionation studies in sorghum have indicated that the distribution of albumin–globulin, prolamin, and glutelin is approximately 15%, 26%, and 44%, respectively, of total nitrogen. The fat content of sorghum grain ranges from 2.1% to 7.6%, crude fiber from 1.0% to 3.4%, and ash from 1.3% to 3.3% ([4]). Starch is the major constituent of grain, accounting for 56%–75% of the total dry matter in the grain [5]. The total soluble sugar content of sorghum grain ranges from 0.7% to 4.2%, whereas reducing sugars range from 0.05% to 0.53% [6]. Another study on the physicochemical characterization of sorghum accessions showed a wide variation in protein (7.99%–17.8%), lipids (2.52%–4.76%), starch (51.88%–85%), and amylose (12.30%–28.38%) content [7]. Linoleic acid (18:2) and oleic acid (18:1) were the major fatty acid constituents of sorghum lipids [7]. The grain is commonly eaten with the testa, which retains the majority of the nutrients. The wide variation in mineral and trace element composition indicates that sorghum is a good source of minerals. The mineral composition, however, is influenced by environmental conditions [8]. Therefore, sorghum can be used as a substitute for wheat flour in bread dough, which is expected to increase the nutritional content of the bread. Sorghum′s sustainability and adaptability to hot, dry climates, together with the unique health‐promoting properties of sorghum flour, make it an attractive ingredient for both the gluten‐free and functional food markets [9]. Sorghum flour can enhance the color intensity and impart chocolate and nutty flavors, as well as graininess and coarseness, to bread [10]. In addition, partial replacement of wheat flour with sorghum flour is promising because sorghum is gluten‐free, richer in dietary fiber and phenolic compounds than wheat, and when incorporated at moderate levels (10%–20%) into wheat–sorghum composite flours, it can yield breads with acceptable technological and sensory quality [11, 12].

Based on several recent studies, sorghum offers multiple advantages over wheat in terms of nutrition, health benefits, and sustainability. Sorghum contains bioactive compounds such as phenolics, flavonoids, and 3‐deoxyanthocyanidins, which contribute to higher antioxidant activity than wheat, making it a promising functional food ingredient [13, 14]. Moreover, sorghum is naturally gluten‐free, providing an ideal alternative for individuals with celiac disease or gluten sensitivity [14]. Agronomically, sorghum exhibits high adaptability to hot and dry climates, efficient water utilization, and strong growth performance across various regions in Indonesia, including marginal lands less suitable for wheat cultivation [15, 16]. Research conducted in Indonesia also shows that sorghum is easy to cultivate and is being promoted nationally as a sustainable local crop supporting food security and carbohydrate diversification [16, 17]. Overall, these characteristics suggest that sorghum has considerable potential as a partial wheat substitute in bread formulations, particularly for improving nutritional quality while supporting the use of local cereal resources.

Dadih is a fermented food produced from buffalo milk and fermented by various lactic acid bacteria (LAB). LAB isolates of dadih consist of three genera, namely, *Lactococcus*, *Lactobacillus*, and *Pediococcus* [18]. LAB can be used as a probiotic because it can survive in the human cecum and has the ability to adhere to the intestine [19]. The total LAB count in dadih reaches up to 10^8^ CFU/mL [20]. LAB has been shown to have antidiabetic, antiobesity, antihypertensive, and immunostimulatory effects [21, 22]. The large number of species isolated from dadih promotes the production of a more acidic product. LAB also produces bioactive compounds that enhance the product′s nutritional value and flavor. Dadih can be processed into a range of products, including raw materials or fortified to improve product quality [23–26].

Traditionally, dadih has been consumed fresh with the addition of onion and chili. Consumer perceptions of traditional products, including dadih, are more favorable [27]. The sensory characteristics of dadih should be mildly sour, white to light cream in color, and tofu‐like in texture. Processing of dadih affects consumer preference and can convert carbohydrates into lactic acid and other metabolites [28]. Similar to yogurt, dadih contains nutritional components. Yogurt provides high vitamin A, riboflavin, calcium, magnesium, iron, zinc, iodine, and selenium relative to the recommended intake for children [29]. Besides, yogurt contains protein, lipids, and LAB [30]. Dadih is a traditional food of the Minangkabau ethnic group from West Sumatra. In the past, it was consumed as a side dish with rice. However, advances in research and scientific knowledge have led to various developments in dadih processing, including its transformation into dadih vla. The continuous development of dadih is also related to efforts to increase the income of buffalo farmers in West Sumatra and to preserve the cultivation of local foods. Therefore, dadih′s sensory preference and nutrients for other products should be analyzed.

Dadih vla is a dairy sauce. Cheese sauce has been shown by Kůrová et al. [31] to exhibit different characteristics following the addition of polysaccharides and homogenization. However, the characteristics of dadih vla have not been evaluated in previous studies. Therefore, this study was aimed at analyzing sensory preference and the nutrient content of dadih vla.

## 2. Materials and Methods

The study employed a functional randomized design (FRD), an experimental research method used to determine the effect of a treatment on a measurable variable. The design comprised two components: sorghum substitution in the sweet bread formula and strawberry addition in the dadih vla formula.

### 2.1. Materials

#### 2.1.1. Sorghum‐Substituted Bread

**Figure 1:**
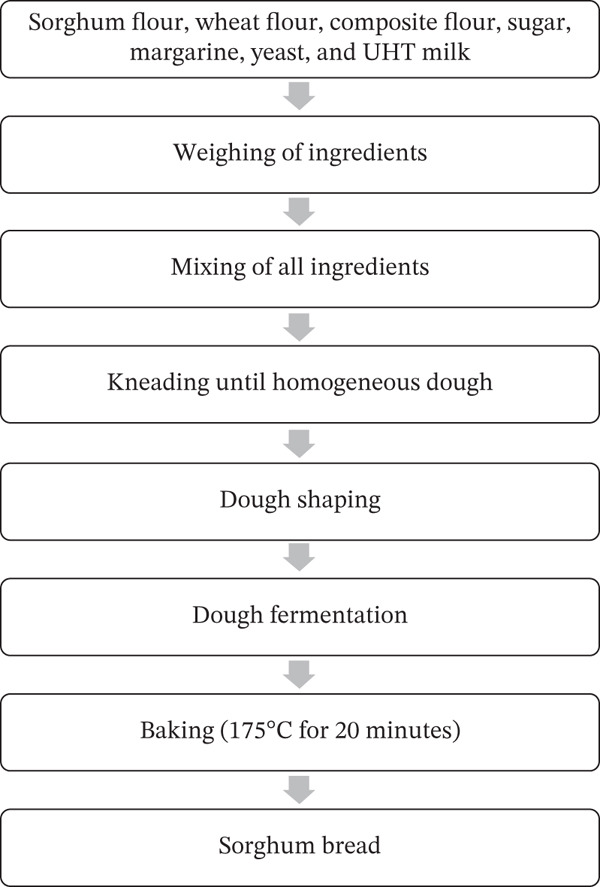
Flowchart of sorghum bread preparation.

The ingredients used to prepare the bread included sorghum flour, wheat flour, composite flour (red bean, soybean, and corn flours), sugar, margarine, yeast, and UHT milk. The composite flour was prepared from locally sourced ingredients, namely, corn, red beans, and soybeans, in a 1:1:1 ratio. The preparation of sorghum‐substituted bread is shown in Figure 1.

#### 2.1.2. Strawberry Dadih Vla

**Figure 2:**
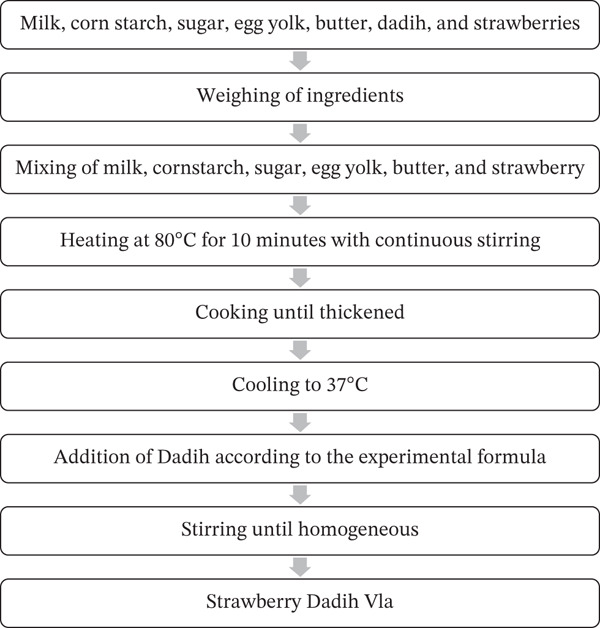
Flowchart of strawberry dadih vla preparation.

Dadih was collected from Agam District, West Sumatra. It was prepared by fermenting pasteurized buffalo milk in bamboo at room temperature for 48 h. The ingredients used to produce strawberry dadih vla were milk, corn starch, sugar, egg yolk, butter, dadih, and strawberries to enhance antioxidant content. All samples were then evaluated using hedonic and hedonic quality tests to determine the best treatment based on sensory parameters. Subsequently, the selected sample was subjected to proximate analysis. The preparation of strawberry dadih vla is shown in Figure 2.

### 2.2. Method

#### 2.2.1. Formulation

Sorghum flour, characterized by its relatively high protein and micronutrient content, was incorporated into the bread formulations at substitution levels of 0% (F0), 5% (F1), and 10% (F2) of the total flour weight. The strawberry addition factor consisted of three concentrations: 0% (F0), 15% (F1), and 20% (F2).

#### 2.2.2. Physicochemical Properties

Physicochemical analyses were conducted to assess water, ash, fat, protein, carbohydrate, and vitamin C contents, as well as pH and color parameters using the Hunter Lab and CIE Lab systems. These tests aimed to determine the physicochemical composition and nutritional properties of the samples. All analyses were performed in triplicate at the Department of Food Technology and Agricultural Products, Faculty of Agricultural Technology, Andalas University.

##### 2.2.2.1. Proximate Analysis

Proximate analysis was performed to determine the major components of the sample, namely, moisture, ash, protein, fat, carbohydrate, and amino acids (AAs). The analysis was conducted in accordance with the Indonesian National Standard (SNI). Moisture content was determined using the gravimetric method based on SNI 01‐2891‐1992, in which the sample was dried at a specified temperature until a constant weight was obtained [32]. Ash content was determined by incinerating the sample at 550°C until a constant weight of ash was obtained, in accordance with SNI 01‐2891‐1992. Fat content was measured by the gravimetric method using an appropriate solvent, also following SNI 01‐2891‐1992. Protein content was analyzed using the Kjeldahl method, in accordance with AOAC 2001 and SNI procedures. This method involves determining total nitrogen and converting it to protein content. Carbohydrate content was calculated by difference, by subtracting the percentages of moisture, ash, protein, and fat from 100%.

The AAs in the selected sample were extracted using the UPLC method (18‐5‐17/MU/SMM‐SIG; UPLC‐PDA). The AAs were derivatized with 6‐aminoquinolyl‐N‐hydroxysuccinimidyl carbamate using the Waters AccQ‐Fluor reagent kit and analyzed by UPLC (Waters 2695 Separation Module, Waters 2475 Multi‐Fluorescence Detector, and Waters AccQ‐Tag AA analysis column). AA content was expressed as milligrams per kilogram of dry matter.

##### 2.2.2.2. Vitamin C

Vitamin C was determined using the iodometric titrimetric method in accordance with SNI 01‐2891‐1992 [32]. The sample was homogenized and prepared as a solution, then titrated with a standard iodometric solution. Vitamin C concentration was calculated based on the volume of titrant used. The analysis was performed using laboratory procedures validated according to international standards.

##### 2.2.2.3. pH

Sample pH was measured using a digital pH meter. Before measurement, the sample was diluted with distilled water at a ratio of 1:10 (sample:water) and then measured at room temperature (25°C).

##### 2.2.2.4. Color

Sample color analysis was conducted using the Hunter Lab Colorimeter system, which measures color parameters using the CIE Lab model. Color was measured by three main parameters: *L* (lightness), which describes the brightness of the sample; *a* for red–green color intensity; and *b* for yellow–blue color intensity. In addition, the b/a ratio (yellow‐to‐red ratio) and hue angle (dominant color) were calculated using these two systems. Measurements were taken at three different points on each sample to obtain representative results. The data were used to analyze color differences resulting from sorghum flour substitution and strawberry addition, as well as to evaluate their effects on the overall color of the product.

#### 2.2.3. Sensory Properties

The sensory evaluation was conducted using a hedonic scoring test to assess sample acceptability. The scoring criteria were as follows: *dislike extremely* (1), *dislike very much* (2), *dislike moderately* (3), *dislike slightly* (4), *neither like nor dislike* (5), *like slightly* (6), *like moderately* (7), *like very much* (8), and *like extremely* (9). The sensory attributes evaluated were color, aroma, taste, and texture. Color quality ranged from pale yellow to white, aroma and taste ranged from less sour to sour, and texture ranged from watery to thick.

The panelists in this test consisted of 30 semitrained individuals, including students from the Master′s Program in Nutrition Science and the Agricultural Products Technology Program at Andalas University. They were asked to evaluate the color, aroma, taste, texture, aftertaste, and overall acceptability of the sample using a 9‐point scale, with ratings ranging from *dislike extremely* (1), *dislike very much* (2), *dislike moderately* (3), *dislike slightly* (4), *neither like nor dislike* (5), *like slightly* (6), *like moderately* (7), *like very much* (8), to *like extremely* (9).

#### 2.2.4. Antioxidant Activity

The antioxidant activity of the selected sample was evaluated using method 18‐9‐97/MU (UV–Vis spectrophotometry). Antioxidant activity was determined using the DPPH method in accordance with SNI 8623‐2018. In this assay, the purple DPPH solution turns pale yellow upon reaction with antioxidant compounds. The resulting color change was measured using a spectrophotometer at approximately 517 nm. Based on measurements at various concentrations, the IC_50_ value was calculated as the concentration of the sample required to inhibit 50% of DPPH free radicals.

#### 2.2.5. Data Analysis

Data obtained from all physicochemical and sensory analyses are presented in the form of average values along with standard deviations. Data were analyzed using two‐way ANOVA with SPSS software to test differences between treatments (substitution of sorghum flour and addition of strawberries). If a significant difference is found (*p* value < 0.05), a further test is carried out using the Duncan test to determine which treatment pairs are significantly different. All data were analyzed in triplicate to ensure the validity of the results.

## 3. Results and Discussion

### 3.1. Physicochemical Properties

#### 3.1.1. Moisture

The moisture content of sorghum‐substituted bread samples with strawberry vla filling in this study ranged from 37.13% to 39.15% (Table 1). The results of the two‐way ANOVA analysis indicate that there is a significant difference (*p* < 0.05) in the water content of the bread samples, which is caused independently by both the sorghum substitution treatment and the addition of strawberries (Table 1). Referring to Duncan′s test results, bread that was substituted for sorghum and added strawberry vla at all concentrations had a significantly higher water content compared to bread without sorghum substitution or without the addition of vla. The moisture content increased in the vla proportion of bread due to the high‐water content in strawberry vla. The increase in moisture content may also be explained by the water‐binding capacity of dietary fiber present in sorghum flour and pectin naturally found in strawberries. These components retain water within the bread matrix during baking, thereby reducing moisture loss and producing a softer crumb.

**Table 1:** Moisture content of sorghum substitute bread with strawberry dadih vla.

Sorghum substitute bread (%)	Vla strawberry (%)		
	0	15	20
0	37.13 ± 0.29^Bb^	38.24 ± 0.67^Ba^	37.84 ± 0.60^Ba^
5	38.20 ± 0.15^Ab^	39.14 ± 0.49^Aa^	38.75 ± 0.61^Aa^
10	37.50 ± 0.39^Ab^	38.93 ± 0.87^Aa^	39.15 ± 0.38^Aa^

*Note:* Different lowercase letters in the same row indicate significant differences (*p* < 0.05) based on the difference in strawberry vla. Different uppercase letters in the same column indicate significant differences (*p* < 0.05) based on differences in sorghum substitute bread—further test using Duncan.

#### 3.1.2. Carbohydrate

The carbohydrate content of sorghum‐substituted bread samples with strawberry vla filling in this study ranged from 45.78% to 51.73% (Table 2). The two‐way ANOVA results indicated significant differences in the carbohydrate content of the bread samples (*p* < 0.05) due to both sorghum substitution and strawberry vla addition (Table 2). Duncan′s post hoc test showed that bread formulated with 10% sorghum and 15% strawberry vla had significantly lower carbohydrate content than the other formulations. These findings suggest that sorghum substitution and strawberry vla addition can reduce the carbohydrate content of bread. The reduction in carbohydrate content is likely attributable to the proportional increase in protein, fat, and moisture fractions following sorghum substitution and strawberry vla incorporation, as carbohydrate values were calculated by difference rather than directly measured.

**Table 2:** Carbohydrate content of sorghum substitute bread with strawberry dadih vla.

Sorghum substitute bread (%)	Vla strawberry (%)		
	0	15	20
0	51.73 ± 0.13^Aa^	48.55 ± 1.24^Ac^	49.73 ± 1.51^Ab^
5	51.16 ± 0.26^Aa^	48.51 ± 0.38^Ac^	49.74 ± 0.49^Ab^
10	48.32 ± 0.55^Ba^	45.78 ± 1.08^Bc^	47.85 ± 0.18^Bb^

*Note:* Different lowercase letters in the same row indicate significant differences (*p* < 0.05) based on the difference in strawberry vla. Different uppercase letters in the same column indicate significant differences (*p* < 0.05) based on differences in sorghum substitute bread—further test using Duncan.

#### 3.1.3. Ash, Protein, Fat, Crude Fiber, and Vitamin C

The ash content of all sorghum‐substituted bread samples with strawberry filling ranged from 0.68% to 0.98% and did not differ significantly (*p* > 0.05) (Table 3). The protein content ranged from 3.85% to 6.15%. Two‐way ANOVA indicated a significant interaction (*p* < 0.05) between sorghum substitution and strawberry vla addition on protein content (Table 3). Bread with sorghum substitution and varying levels of vla addition had significantly higher protein content than bread without sorghum substitution. Protein content increased significantly with higher levels of sorghum incorporation, likely due to the higher protein content of sorghum (11%) compared with wheat (9%) [33].

**Table 3:** Ash, protein, fat, crude fiber, and vitamin C content of sorghum substitute bread with strawberry dadih vla.

Treatment	Parameter				
	Ash	Protein	Fat	Crude fiber	Vitamin C
0% sorghum, 0% strawberry	0.88 ± 0.26^a^	3.85 ± 0.06^b^	6.40 ± 0.24^c^	1.18 ± 0.11^c^	58.44 ± 12.72^abc^
0% sorghum, 15% strawberry	0.84 ± 0.07^a^	4.04 ± 0.26^b^	8.33 ± 0.71^a^	1.54 ± 0.32^bc^	21.97 ± 0.07^f^
0% sorghum, 20% strawberry	0.71 ± 0.07^a^	4.04 ± 0.28^b^	7.67 ± 1.22^a^	2.19 ± 0.12^a^	29.25 ± 12.70^ef^
5% sorghum, 0% strawberry	0.68 ± 0.19^a^	4.45 ± 0.29^b^	5.51 ± 0.75^cd^	1.62 ± 0.28^bc^	51.11 ± 12.64^bcd^
5% sorghum, 15% strawberry	0.76 ± 0.05^a^	5.85 ± 0.59^a^	5.74 ± 0.18^c^	1.81 ± 0.38^ab^	43.89 ± 0.04^cde^
5% sorghum, 20% strawberry	0.98 ± 0.35^a^	6.02 ± 0.38^a^	4.49 ± 0.59^d^	1.31 ± 0.14^c^	51.20 ± 12.81^bcd^
10% sorghum, 0% strawberry	0.88 ± 0.03^a^	5.72 ± 0.29^a^	7.57 ± 0.28^ab^	1.27 ± 0.17^c^	36.60 ± 12.69^def^
10% sorghum, 15% strawberry	0.87 ± 0.04^a^	6.15 ± 0.64^a^	8.26 ± 0.57^a^	1.15 ± 0.15^c^	73.19 ± 12.71^a^
10% sorghum, 20% strawberry	0.77 ± 0.12^a^	5.68 ± 0.24^a^	6.54 ± 0.02^bc^	1.32 ± 0.35^c^	65.74 ± 0.17^ab^

*Note:* Superscript letters indicate significant differences (*p* < 0.05).

The fat content of all samples ranged from 1.15% to 8.33%. The results of the two‐way ANOVA analysis showed that there was a significant difference (*p* < 0.05) in the fat content of the samples, which was caused by the interaction between the sorghum substitution treatment and the addition of strawberry vla (Table 3). In each sample of sorghum substitute bread, there was a tendency for the fat content of the bread to be higher with the addition of 15% strawberry vla compared to other strawberry vla formulas.

The crude fiber content of the bread samples ranged from 1.15% to 2.19%. Bread without sorghum substitution and with 20% strawberry vla had the highest crude fiber content, which was significantly greater than that of the other formulas. Vitamin C content ranged from 21.97% to 73.19%. Bread formulated with 10% sorghum substitution and 20% strawberry vla had significantly higher vitamin C content than the other bread formulas.

#### 3.1.4. Physical Properties

The pH of the bread samples ranged from 4.83 to 5.38 and did not differ significantly (*p* > 0.05) (Table 4). The lightness (*L*), redness (*a*), and yellowness (*b*) values ranged from 56.87 to 69.48, 1.19 to 6.12, and 13.27 to 19.85, respectively. Two‐way ANOVA showed that the interaction between sorghum substitution and strawberry vla addition significantly affected lightness (*L*) and yellowness (*b*) (*p* < 0.05), whereas redness (*a*) was not significantly affected (*p* > 0.05) (Table 4).

**Table 4:** Physical characteristics of sorghum substitute bread with strawberry dadih vla.

Treatment		Parameter					
	pH	*L*	*a*	*b*	*b*/*a*	Hue	*Δ* *E*
0% sorghum, 0% strawberry	4.99 ± 0.64	60.75 ± 10.74^ab^	2.52 ± 1.50	15.66 ± 1.09	7.71 ± 3.78	80.67 ± 6.11	11.47 ± 9.32
0% sorghum, 15% strawberry	5.24 ± 0.10	69.48 ± 8.02	1.37 ± 0.69	17.95 ± 0.76	15.19 ± 6.37	85.55 ± 2.45	7.51 ± 2.66
0% sorghum, 20% strawberry	5.02 ± 0.39	49.00 ± 4.55	1.19 ± 0.43	13.27 ± 1.11	12.75 ± 6.85	84.73 ± 2.23	22.40 ± 4.52
5% sorghum, 0% strawberry	5.38 ± 0.24	64.15 ± 6.48	2.13 ± 0.28	15.93 ± 1.34	7.53 ± 0.40	82.42 ± 0.39	8.13 ± 5.75
5% sorghum, 15% strawberry	4.89 ± 0.45	56.87 ± 8.23	5.60 ± 2.97	16.97 ± 2.45	4.08 ± 3.15	71.59 ± 9.56	8.37 ± 6.97
5% sorghum, 20% strawberry	4.83 ± 0.56	63.41 ± 7.64	4.92 ± 0.49	18.87 ± 2.05	6.35 ± 3.77	76.69 ± 11.48	13.93 ± 8.02
10% sorghum, 0% strawberry	5.03 ± 0.39	60.18 ± 1.17	4.95 ± 2.88	19.46 ± 2.68	5.42 ± 3.85	76.44 ± 6.79	10.38 ± 0.56
10% sorghum, 15% strawberry	5.26 ± 0.34	57.66 ± 1.04	6.12 ± 0.30	19.85 ± 0.53	3.24 ± 0.07	72.85 ± 0.38	12.39 ± 1.06
10% sorghum, 20% strawberry	5.06 ± 0.41	57.99 ± 0.84	3.25 ± 0.67	17.09 ± 0.85	5.38 ± 0.84	79.29 ± 1.79	12.50 ± 0.98

*Note:* Superscript letters indicate significant differences (*p* < 0.05).

The substitution of sorghum flour in bread significantly affected the color parameters, particularly lightness (*L*), yellowness (*b*), and redness (*a*). Increasing the proportion of sorghum flour tended to decrease lightness (*L*) and yellowness (*b*) while increasing redness (*a*). These findings are consistent with those of Adzqia et al. [10] and Aguiar et al. [34]. These color changes are primarily attributable to the natural pigments present in the sorghum pericarp, which play a more prominent role in determining the final product color than Maillard reactions and caramelization during processing [35].

The significant correlation between the color of sorghum flour and that of the mixture indicates that the color of the raw material strongly determines the visual characteristics of the final product [36]. This finding is consistent with consumer preferences for darker colored products, which are often associated with health benefits. In some countries, such as Germany and those in Eastern Europe, darker colors are perceived as healthier. This perception is further supported by growing consumer interest in dark‐colored foods, which are believed to contain antioxidants and other bioactive compounds with potential health benefits [35, 36]. Thus, sorghum flour can enhance color and serve as an additional advantage, not only in terms of visual characteristics but also in meeting market preferences for products made from natural ingredients and associated with high health value.

### 3.2. Sensory Properties

The acceptability scores of the bread samples based on color, aroma, taste, and aftertaste ranged from 6.68 to 7.07, 6.32 to 7.07, 5.58 to 6.32, and 5.60 to 6.40, respectively, and did not differ significantly (*p* > 0.05) (Table 5). Acceptability scores for texture and overall liking ranged from 5.68 to 7.04 and from 6.08 to 6.88, respectively. Two‐way ANOVA showed that texture acceptability differed significantly (*p* < 0.05) as a result of both sorghum substitution and strawberry vla addition (Table 6). Duncan′s post hoc test indicated that bread without sorghum substitution and with 15% strawberry vla had significantly higher texture acceptability than the other formulations. Two‐way ANOVA for overall acceptability also showed a significant effect (*p* < 0.05) of sorghum substitution (Table 7). Panelists significantly preferred the texture of bread without sorghum substitution.

**Table 5:** Sensory acceptance according to color, aroma, taste, and aftertaste of sorghum substitute bread with strawberry dadih vla.

Treatment	Parameter			
	Color	Aroma	Taste	Aftertaste
0% sorghum, 0% strawberry	7.07 ± 1.64	7.07 ± 1.29	6.11 ± 1.55	6.27 ± 1.61
0% sorghum, 15% strawberry	6.84 ± 1.67	6.32 ± 1.65	5.68 ± 1.67	5.60 ± 1.82
0% sorghum, 20% strawberry	7.00 ± 1.53	6.64 ± 1.89	6.08 ± 1.70	6.00 ± 1.61
5% sorghum, 0% strawberry	6.72 ± 1.72	6.80 ± 1.35	6.48 ± 1.50	6.40 ± 1.41
5% sorghum, 15% strawberry	6.68 ± 1.60	6.68 ± 1.49	6.12 ± 1.16	6.12 ± 1.42
5% sorghum, 20% strawberry	6.76 ± 0.145	6.36 ± 1.84	5.68 ± 1.57	5.84 ± 1.46
10% sorghum, 0% strawberry	6.88 ± 1.51	6.72 ± 1.06	6.08 ± 1.49	5.76 ± 1.58
10% sorghum, 15% strawberry	6.72 ± 1.62	6.60 ± 1.47	6.32 ± 1.49	5.88 ± 1.72
10% sorghum, 20% strawberry	6.68 ± 1.34	6.68 ± 1.31	5.68 ± 1.28	5.68 ± 1.34

**Table 6:** Texture sensory acceptance of sorghum substitute bread with strawberry dadih vla.

Sorghum substitute bread (%)	Vla strawberry (%)		
	0	15	20
0	7.04 ± 1.61	6.84 ± 1.65	6.20 ± 1.84
5	5.80 ± 1.75	6.48 ± 1.23	5.68 ± 1.84
10	6.12 ± 1.36	6.24 ± 1.64	5.88 ± 1.33

**Table 7:** Overall sensory acceptance of sorghum substitute bread with strawberry dadih vla.

Sorghum substitute bread (%)	Vla strawberry (%)		
	0	15	20
0	6.65 ± 1.41	6.08 ± 1.75	6.28 ± 1.51
5	6.88 ± 1.16	6.36 ± 0.99	6.08 ± 1.28
10	6.48 ± 1.16	6.24 ± 1.50	6.16 ± 0.98

According to the sensory analysis, the acceptability scores of bread formulated with sorghum and strawberry vla did not differ significantly for color, aroma, taste, or aftertaste (*p* > 0.05). However, texture and overall acceptability differed significantly (*p* < 0.05). Texture acceptability was independently affected by sorghum substitution and strawberry vla addition. In particular, bread without sorghum substitution and with 15% strawberry vla received the highest texture score, indicating that strawberry vla addition positively influenced bread texture. Sorghum flour contains no gluten‐forming proteins. Consequently, increasing substitution levels diluted the gluten network, resulting in reduced gas retention, denser crumb structure, and lower texture acceptability.

However, the overall acceptability test indicated that sorghum substitution had a significant effect (*p* < 0.05), with panelists preferring bread without sorghum in terms of texture. Although sorghum increases the compositional diversity of bread, these findings suggest that strawberry vla addition may improve textural quality, whereas sorghum substitution may be less favorable overall.

In general, the addition of strawberry vla has a more positive impact on consumer acceptance, especially in bread formulations containing 15% strawberry vla. This suggests that, although sorghum has the potential to increase the nutritional value of bread, the addition of strawberry vla has a greater effect on consumer preference, particularly in terms of the bread′s texture and taste.

On the other hand, based on the panelists′ overall perceptions, the addition of sorghum and vla can make the bread browner in color, whereas vla contributes to a brighter and more appealing appearance. The aroma did not differ substantially from that of bread made from other grains. The mean taste scores of bread with added strawberry curd vla were nearly identical across all formulations, indicating that vla did not significantly alter the flavor profile. However, the aftertaste was perceived increasingly unfavorably as the proportion of sorghum substitution increased, which is consistent with [37], who reported that higher levels of sorghum flour substitution in bread reduced taste and texture preferences. In addition, the incorporation of sorghum flour and curd flour tended to lower panelists′ preference scores for bread color. Color is an important determinant of bread quality because it is one of the first attributes that attracts consumer attention. Mixing sorghum flour with other flours, such as wheat flour, can produce bread with a darker crumb and crust color, as reported in previous studies [38].

### 3.3. AA Profile and Antioxidant Activity (IC_50_)

Table 8 presents the AA composition of bread containing 5% sorghum substitution and 15% strawberry vla. The most abundant AA in this formulation was glutamate, followed by proline, aspartic acid, serine, histidine, phenylalanine, glycine, arginine, valine, alanine, threonine, isoleucine, tyrosine, tryptophan, cystine, and methionine. The highest glutamate content was observed in the bread with 5% sorghum substitution and 15% strawberry vla. This was mainly attributable to the high glutamate content of sorghum (21.11 g/100 g protein or 17.50–23.42 g/100 g sorghum) [39, 40], as well as the high glutamate content of dadih (15.6–16.8 mg/g) [41]. Higher proline levels were also found in the bread with 5% sorghum substitution and 15% strawberry vla, due to the high proline content of sorghum (6.66–9.4 g/100 g sorghum) [39] and dadih (6.3–6.9 mg/g) [41]. The other substitution breads exhibited lower glutamate, proline, and histidine contents than the bread with 5% sorghum substitution and 15% strawberry vla [42]. These elevated values in the formulated bread were primarily due to the high AA contents of sorghum and dadih.

**Table 8:** Amino acid profile and antioxidant activity of sorghum substitute bread 5% with strawberry vla 15%.

Parameters	5% sorghum, 15% strawberry
*Amino acid*	
Alanine (mg/kg)	3240.03 ± 11.19
Arginine (mg/kg)	3344.91 ± 10.97
Aspartic acid (mg/kg)	5351.95 ± 6.92
Glycine (mg/kg)	3367.87 ± 4.31
Glutamate (mg/kg)	20,126.48 ± 19.38
Histidine (mg/kg)	4343.55 ± 10.55
Isoleucine (mg/kg)	2569.12 ± 6.76
Cystine (mg/kg)	492.69 ± 1.32
Methionine (mg/kg)	36.06 ± 0.01
Tryptophan (mg/kg)	860.52 ± 3.77
Valine (mg/kg)	3247.21 ± 10.04
Phenylalanine (mg/kg)	3695.29 ± 10.62
Proline (mg/kg)	7567.04 ± 34.05
Serine (mg/kg)	4512.95 ± 17.24
Threonine (mg/kg)	2675.36 ± 4.51
Tyrosine (mg/kg)	1319.57 ± 12.81
*Antioxidant activity (μg/mL)*	93,667.44 ± 1.27

The antioxidant activity of the bread containing 5% sorghum substitution and 15% strawberry vla is presented in Table 8. The antioxidant activity of the sample is attributable to sorghum, which is recognized as one of the cereals with the greatest antioxidant potential [1]. This strong antioxidant activity is associated with the presence of phenolic compounds and flavonoids [2]. Compared with similar cereal‐based products reported in the literature, the IC_50_ values in this study are relatively higher, indicating that the antioxidant potential of the sorghum–strawberry bread is moderate to low. Nevertheless, the presence of phenolic compounds and flavonoids may still contribute to its functional properties [43]. Sorghum is a plant in which phenolic compounds are predominantly composed of phenolic acids, 3‐deoxyanthocyanidins, and condensed tannins. Phenolic acids, including protocatechuic acid (150.3–178.2 mg/g), ferulic acid (120.5–173.5 mg/g), gallic acid (14.8–20.0 mg/g), and vanillic acid (15.4–23.4 mg/g), are abundant in sorghum and have been shown to exhibit anti‐inflammatory and antioxidant activities, as well as additional health benefits [44, 45]. Meanwhile, apigeninidin, apigeninidin 5‐glucoside, luteolinidin, luteolinidin 5‐glucoside, and other 3‐deoxyanthocyanidins are the main flavonoids present in sorghum grain [1]. Flavonoids constitute an important class of phenolic compounds in sorghum and are associated with health‐promoting effects. Therefore, sorghum‐substituted bread with antioxidant activity may possess potential health benefits. Phenolic acids and flavonoids donate hydrogen atoms or electrons to neutralize DPPH free radicals, thereby interrupting radical chain reactions. Although the measured IC_50_ indicated only moderate antioxidant activity, the presence of these compounds suggests that the product retained a proportion of sorghum‐derived bioactive constituents after processing.

Overall, the present findings demonstrate that moderate sorghum substitution (5%) combined with 15% strawberry dadih vla provides a balanced compromise between nutritional enhancement and consumer acceptability. Although higher substitution levels further improved several nutritional parameters, they tended to reduce texture and overall sensory acceptance due to gluten dilution and changes in crumb structure. These results indicate that moderate substitution levels may represent the most practical formulation for future development of sorghum‐based functional bakery products. Nevertheless, further studies evaluating phenolic compounds, shelf life, glycemic response, and in vivo health effects are required before broader functional claims can be established.

## 4. Conclusions

Bread substituted with sorghum and filled with strawberry vla showed significant differences in water, carbohydrate, protein, fat, crude fiber, and vitamin C contents compared with the control bread. The water content of the sorghum‐substituted bread with strawberry vla was higher, whereas carbohydrate content tended to be lower in bread containing 10% sorghum and 15% strawberry vla. Sorghum substitution also increased protein, fat, and vitamin C contents, particularly in bread containing 10% sorghum and 20% strawberry vla. In terms of physical properties, these breads showed significant differences in lightness and yellowness but no significant differences in pH or redness. Sensory evaluation showed that consumer preference for the texture of bread containing 15% strawberry vla was higher; however, overall, there were no significant differences in acceptance of taste, aroma, or appearance. The AA composition of bread with 5% sorghum substitution and 15% strawberry vla was dominated by glutamate, proline, and histidine. Sorghum and dadih also contributed to the antioxidant activity of the bread.

Substituting wheat flour with sorghum flour and incorporating strawberry vla significantly affected the nutritional and physicochemical properties of bread. Sorghum substitution increased the protein, fat, crude fiber, and vitamin C contents while slightly reducing carbohydrate levels. The addition of strawberry vla also increased moisture and vitamin C contents, thereby improving the nutritional quality of the bread. In terms of physical properties, sorghum‐based breads exhibited significant differences in lightness and yellowness, whereas pH and redness were comparable to those of the control. Sensory evaluation showed that bread containing 15% strawberry vla received the highest texture scores, whereas overall acceptance of taste, aroma, and appearance remained satisfactory across formulations. The AA profile of bread containing 5% sorghum and 15% strawberry vla was dominated by glutamate, proline, and histidine. Together with its higher antioxidant activity, these findings support the feasibility of using sorghum as a partial wheat flour substitute capable of improving several nutritional characteristics of bread.

## Funding

This study was funded by the Lembaga Penelitian dan Pengabdian kepada Masyarakat, Universitas Andalas (24/UN16.19/PT.01.03/RKI/2024).

## Conflicts of Interest

The authors declare no conflicts of interest.

## Data Availability

The data that support the findings of this study are available from the corresponding author upon reasonable request.
